# Genetic Diversity of Bile Salt Hydrolases Among Human Intestinal Bifidobacteria

**DOI:** 10.1007/s00284-013-0362-1

**Published:** 2013-04-17

**Authors:** Piotr Jarocki, Zdzisław Targoński

**Affiliations:** Department of Biotechnology, Human Nutrition and Food Commodities, University of Life Sciences in Lublin, 8 Skromna St., 20-704 Lublin, Poland

## Abstract

This study analyzes the application of degenerative primers for the screening of bile salt hydrolase-encoding genes (*bsh*) in various intestinal bifidobacteria. In the first stage, the design and evaluation of the universal PCR primers for amplifying the partial coding sequence of bile salt hydrolase in bifidobacteria were performed. The amplified *bsh* gene fragments were sequenced and the obtained sequences were compared to the *bsh* genes present in GenBank. The determined results showed the utility of the designed PCR primers for the amplification of partial gene encoding bile salt hydrolase in different intestinal bifidobacteria. Moreover, sequence analysis revealed that bile salt hydrolase-encoding genes may be used as valuable molecular markers for phylogenetic studies and identification of even closely related members of the genus *Bifidobacterium*.

## Introduction

Members of the genus *Bifidobacterium* are anaerobic Gram-positive *Actinobacteria*, which are commonly found in the gastrointestinal tract (GIT) of humans and animals [21, 22]. Numerous studies have shown that the presence of the bifidobacteria in the human gut is associated with many health-promoting effects, such as immunomodulation, prevention of diarrhea, reduction of pathogens and toxic compounds, decrease of lactose intolerance, and stabilization of the state of the intestine [2, 3, 6, 11]. Because of this, they are frequently used as food additives in the dairy industry and therapeutic agents in some probiotic pharmaceuticals.

In previous research, various molecular methods were used for detection and specific identification of *Bifidobacterium* strains [10, 23]. Among them, a sequence analysis of 16S rDNA has been used widely for both the preparation of species-specific PCR primers and bacterial-phylogeny analysis [13, 14]. However, because some bifidobacterial species showed a high degree of similarity to this gene sequence, alternative molecular markers were tested as well as applied for rapid identification, and a phylogenetic relationship study between different members of the genus *Bifidobacterium* [5, 22].

It has been suggested previously that genetic analysis of bile salt hydrolases may provide important information about the phylogeny and the genetic diversity of the genus *Bifidobacterium* [7]. In this study, we present the application of degenerative primers for the screening of *bsh* genes in various human intestinal bifidobacteria. In the first stage, the design and evaluation of the universal PCR primers for amplifying the partial coding sequence of bile salt hydrolase in different species of *Bifidobacterium* were performed. Next, the amplified gene fragments were sequenced, and the obtained results were then compared with the *bsh* genes present in GenBank. Finally, the expression of bile salt hydrolase genes was tested by the RT-PCR detection of internal fragments of the analyzed *bsh* genes.

## Materials and Methods

### Bacterial Strains and Culture Conditions

This study involved twenty-two strains of bifidobacteria (Table 1). All intestinal isolates were cultured in a modified Garche’s medium [4]. BSH-negative strains (*B. asteroides* and *B. coryneforme*) which originated from the hindgut of honeybee, were grown in MRS broth (Oxoid) supplemented with cysteine HCl. The cultures were routinely incubated at 37 °C in an anaerobic jar (Oxoid) for 24 h.

**Table 1:** List of bacterial strains used in this study

Strain	GenBank accession no.
*Bifidobacterium adolescentis* DSM 20083^T^	JX880242
*Bifidobacterium adolescentis* DSM 20086	JX880243
*Bifidobacterium adolescentis* DSM 20087	JQ696811
*Bifidobacterium angulatum* DSM 20098^T^	JX880238
*Bifidobacterium animalis* subps. lactis NRRL B-41405	JQ696813
*Bifidobacterium animalis* subps. animalis NRRL B-41406^T^	JQ696812
*Bifidobacterium asteroides* DSM 20089^T^	–
*Bifidobacterium bifidum* DSM 20456^T^	JQ696814
*Bifidobacterium breve* DSM 20091	JQ696815
*Bifidobacterium breve* NRRL B-41408^T^	JQ696816
*Bifidobacterium catenulatum* DSM 20224	JQ696817
*Bifidobacterium coryneforme* DSM 20216^T^	–
*Bifidobacterium gallicum* DSM 20093^T^	JX880240
*Bifidobacterium longum* subps. infantis ATCC 15697^T^	JQ696818
*Bifidobacterium longum* subps. longum NRRL B-41409^T^	JQ696819
*Bifidobacterium longum* subps. suis NRRL B-41407^T^	JQ696822
*Bifidobacterium pseudocatenulatum* DSM 20439	JQ696820
*Bifidobacterium pseudolongum* subsp. globosum DSM 20092^T^	JX880241
*Bifidobacterium pseudolongum* subsp. pseudolongum DSM 20094	JX880244
*Bifidobacterium pseudolongum* subsp. pseudolongum DSM 20095	JX880245
*Bifidobacterium pseudolongum* subsp. pseudolongum DSM 20099^T^	JQ696821
*Bifidobacterium ruminantium* DSM 6489^T^	JX880239

### Total DNA Extraction and Polymerase Chain Reaction

The isolation of chromosomal DNA from *Bifidobacterium* strains used in this study was carried out from an overnight culture, using the Total DNA Mini Kit (A&A Biotechnology, Poland). Universal primers were designed for the amplification of the partial genes encoding the bile salt hydrolase (Table 2). Each reaction mixture contained 0.25 U of *Taq* DNA polymerase (Fermentas), 200 μM of each deoxynucleoside triphosphate, PCR buffer (Fermentas), 1 or 2 μM of each primer, and 1 μl (10–30 ng) of bacterial DNA in the final volume of 20 μl. Amplification was performed using the LabCycler (SensoQuest, Germany) programmed as follows: 5 min at 94 °C for initial denaturation and 35 cycles of 1 min at 94 °C, an annealing step at 55–60 °C for 1 min, 1 min at 72 °C for extension and 10 min at 72 °C for a final extension. Five stool samples were collected from healthy humans and stored in −20 °C. Genomic DNA from a portion of fecal samples (about 200 mg) was isolated by using a GeneMATRIX Stool DNA Purification Kit (EURx, Poland). Six probiotic products including different kinds of liquid yogurts manufactured by five companies were collected. Next, DNA was extracted directly from 300 mg of each yogurth sample by using a GeneMATRIX Food-Extract DNA Purification Kit (EURx, Poland). Afterward, 1 and 5 μl of the resultant samples were used for PCR screening. The PCR products were analyzed by agarose gel electrophoresis with 1.4 % (w/v) agarose in a Tris–acetate-EDTA buffer (TAE). The gels were stained with ethidium bromide (0.5 μg/ml) and visualized under UV light.

**Table 2:** Sequences of oligonucleotide primers used in this study

Primer	Nucleotide sequence (5′–3′)	Use
Bif-bshA-1F	ATGTGCACWGSYGTYCGTTT	PCR screening, sequencing
Bif-bshB-2F	TTCGGCCGYAAYCTCGAYTG GAG	PCR screening
Bif-bshC-1R	TCGAYGACGATGCTDCG	PCR screening
Bif-bshD-2R	GGYTGRTTGGTVAGCACRTC	PCR screening, sequencing
Bif-bshE-3F	TTCGGCCGYAAYCTYGAYTGG	PCR screening, RT-PCR
Bif-bshF-3R	TCGAYGACGATGSTDCG	PCR screening, RT-PCR

### DNA Sequencing and Sequence Analysis

The amplified *bsh* gene fragments, obtained with Bif-bshA-1F and Bif-bshD-2R primers were purified using ExoSAP-IT (USB) and were subsequently sequenced. The nucleotide sequences were determined using a BigDye Terminator v3.1 Cycle Sequencing Kit (Applied Biosystems) and the capillary sequencing system 3730*xl* DNA Analyzer (Applied Biosystems). The partial gene sequences obtained in this study and those from the databases were analyzed by Clustal [20] and BLAST [1]. The phylogenetic tree was calculated using neighbor joining method [16] with software package MEGA version 4.0 [18]. The determined nucleotide sequences have been deposited in the GenBank database (Table 1).

### Isolation of Bacterial RNA and RT-PCR

For isolation of RNA from bifidobacteria, cells of overnight cultures were collected by centrifugation at 10,000×*g* for 5 min, and then treated with a lysozyme (MP Biomedicals) solution (5 mg/ml) prepared in a TE buffer (10 mM Tris pH 7.5, 1 mM EDTA). The total RNA was isolated with a GeneMATRIX Universal RNA Purification Kit (EURx, Poland) according to the manufacturer’s instructions, and the samples were then treated with DNase (Fermentas) at 37 °C for 30 min. DNase was inactivated by using EDTA and incubated at 65 °C for 10 min. The reverse transcription of isolated RNA samples and PCR of cDNA were performed by using a GeneAmp RNA PCR Kit (Applied Biosystems) as recommended by the manufacturer. The PCR amplification was done with internal primers for the *bsh* genes (Table 2) in a final volume of 20 μl (including 4 μl from the RT reaction) under standard conditions as described above.

## Results and Discussion

### Primer Design and PCR Amplification

**Fig. 1:**
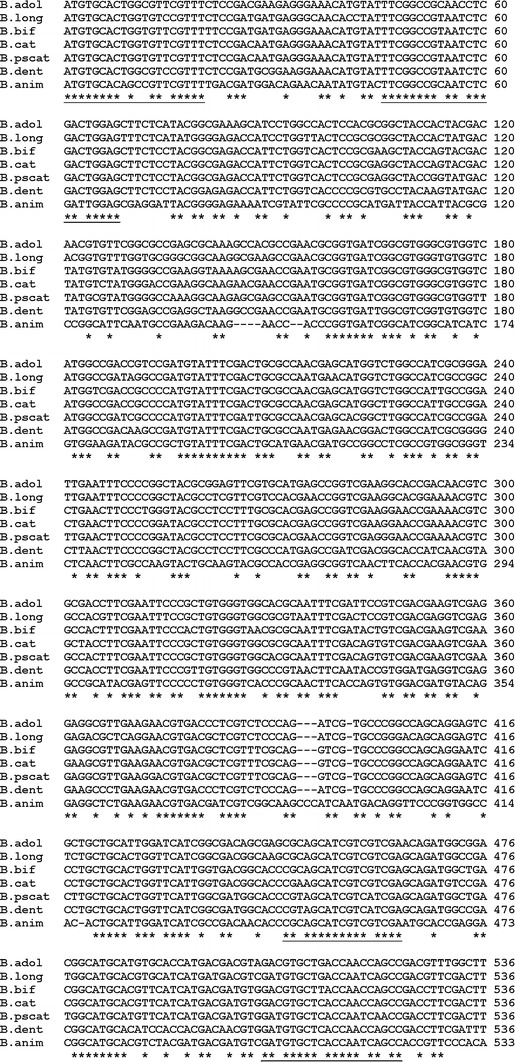
Alignment of partial *bsh* genes from various bifidobacteria and design of universal primers for PCR. Abbreviation for BSHs: *B. adol* *B. adolescentis* ATCC 15703 (AP009256.1), *B. long* *B. longum* SBT2928 (AF148138.1), *B. bif* *B. bifidum* ATCC 29521 (AY604517.1), *B. cat* *B. catenulatum* DSM 16992 (NZ_ABXY01000011.1), *B. pscat* *B. pseudocatenulatum* DSM 20438 (NZ_ABXX02000003.1), *B. dent* *B. dentium* Bd1 (CP001750.1), *B. animalis* subsp. *lactis* KL612 (AY530821.1). *Asterisks* indicate identical nucleotides. Four highly conserved regions of *bsh* genes used for primers design are *underlined*

**Fig. 2:**
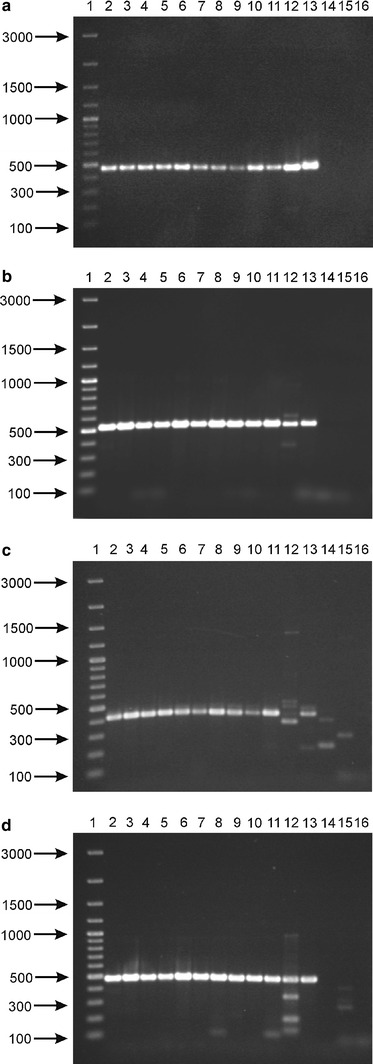
PCR screening of different Bifidobacterium strains with primers: Bif-bshA-1F and Bif-bshC-1R (**a**), Bif-bshA-1F and Bif-bshD-2R (**b**), Bif-bshB-2F and Bif-bshC-1R (**c**), and Bif-bshB-2F and Bif-bshD-2R (**d**). *Lanes: 1* DNA molecular weight marker (in base pairs), *2* *B. adolescentis* DSM 20087, *3* *B. animalis* subps. *lactis* NRRL B-41405, *4* *B. animalis* subps. *animalis* NRRL B-41406, *5* *B. bifidum* DSM 20456, *6* *B. breve* DSM 20091, *7* *B. breve* NRRL B-41408, *8* *B. catenulatum* DSM 20224, *9* *B. longum* subsp. *infantis* ATCC 15697, *10* *B. longum* subsp. *longum* NRRL B-41409, *11* *B. pseudocatenulatum* DSM 20439, *12* *B. pseudolongum* DSM 20099, *13* *B. longum* subsp. *suis* NRRL B-41407, *14* *B. asteroides* DSM 20089, *15* *B. coryneforme* DSM 20216, *16* negative control

Modern molecular techniques based on sequence comparisons of housekeeping genes provide very useful information on the composition and phylogeny of the normal gastrointestinal microbiota. Moreover, a multilocus sequence analysis of conserved genes allows more reliable identification of isolated strains at the genus, species and sometimes even at the strain level. In this study, we analyzed the utility of bile salt hydrolase-encoding genes as genetic markers which can be applied in the specific and sensitive evaluation of the intestinal bifidobacteria. In order to determine whether all the tested strains possess *bsh* gene, the degenerative primers were designed based on comparison of the *bsh* sequences present in GenBank. We aligned four previously described BSH coding genes from four bifidobacterial species: *B. longum* [19], *B*. *bifidum* [9], *B. adolescentis* [7], and *B. animalis* [8]. In addition, three *bsh* sequences for *B. catenulatum*, *B. dentium*, and *B. pseudocatenulatum* originating from whole genome sequences database, were also analyzed (Fig. 1). The performed comparison indicated four highly conserved regions of *bsh* genes for tested bifidobacteria. Two forward primers: Bif-bshA-1F and Bif-bshB-2F with target sites starting at position 1 and 46 (with reference to the *B. bifidum* ATCC 29521 *bsh* gene, GenBank accession number: AY604517) were selected. Two reverse primers, Bif-bshC-1R and Bif-bshD-2R, were designed based on other conserved sites starting at positions 448 and 505, respectively. All primers designed in this study were then tested in polymerase chain reactions with DNA isolated from 14 strains of bifidobacteria. The obtained results demonstrated the utility of these primers for the amplification of *bsh* fragments from different intestinal bifidobacterial species (Fig. 2). The amplification was less efficient for *B. pseudolongum* compared with the other strains. In addition, PCR with the third oligonucleotide pair (Bif-bshB-2F and Bif-bshC-1R) showed that this primer set was not suitable for amplification of the specific fragment of *bsh* of *B. pseudolongum*. In all cases, two BSH-negative reference strains (*B. asteroides* and *B. coryneforme*) were negative in the PCR screening with the above primer sets and gave only weak and unspecific bands.

**Fig. 3:**
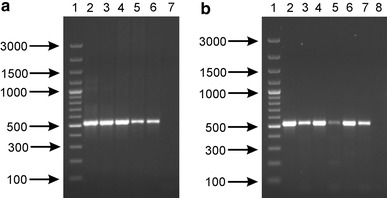
Agarose gel electrophoresis of PCR products obtained with primers Bif-bshA-1F and Bif-bshD-2R for DNA samples extracted from feaces (**a**) and probiotic products (**b**). *Lanes*: **a** *1* DNA molecular weight marker (in base pairs), *2*–*6* genomic DNAs directly extracted from five stool samples, *7* negative control, **b** *1* DNA molecular weight marker, *2–7* genomic DNA directly extracted from six probiotic products, *8* negative control

The utility of designed primers for the detection of BSH-positive *Bifidobacterium* strains was also evaluated using DNA isolated from fecal samples and dairy products. As expected, the PCR screening with primers Bif-bshB-2F and Bif-bshC-1R, showed that the specific products of expected size (524 bp) were obtained for all DNA samples isolated from feces (Fig. 3a). Likewise, all assayed commercial probiotic products also gave positive results (detectable, specific bands) showing that they contain bacterial strains with *bsh* genes and the presumed bile salt activity (Fig. 3b). The obtained results showed that the developed primers can be useful for rapid detection of BSH-positive bifidobacteria and for the monitoring of the occurrence of BSH activity in the analyzed samples.

### Determination and Analysis of the Partial Coding Sequences of Bile Salt Hydrolase

Previously amplified PCR products generated with the Bif-bshA-1F and Bif-bshD-2R primers for 20 bifidobacteria were purified and sequenced. Subsequently, the obtained results were aligned with *bsh* genes present in GenBank. For all tested strains, the amplified DNA fragment contained a partial sequence of *bsh*. In the case of *B. adolescentis* DSM 20087, the generated *bsh* sequence showed a high similarity (more than 99.6 %) with strains of *B. adolscentis* previously described [7]. In addition, all the analyzed *bsh* sequences from this species share more than 99 % similarity with *bsh* sequence obtained for *B. ruminantium*. These results were similar to the recent analysis of 16S rRNA gene sequences, which indicated high relatedness of these two taxa. The obtained sequences revealed significantly lower similarity (below 89 %) with *bsh* genes from other species of *Bifidobacterium*. Likewise, different strains of *B. animalis* subsp. *lactis* were also observed to possess identical *bsh* gene sequences and to share at most 72 % similarity with respect to all of the other members of the analyzed genus. Interestingly, partial *bsh* sequence of *B. animalis* subsp. *animalis* revealed 98.7 % identity with a respective sequence from *B. animalis* subsp. *lactis*. This result confirmed previous findings which indicated that these two taxa should be distinguished and considered as two separate subspecies in *B. animalis* [12].

Comparison of the obtained *bsh* sequence for type strain of *B. bifidum* with other sequences present in GenBank revealed that among the strains of the species *B. bifidum*, the BSH DNA sequence similarities ranged from 97.3 to 99.6 %. In addition, the type strain used in this study revealed a high degree of similarity with *B. catenulatum* DSM 20224 (95 %) and *B. pseudocatenulatum* DSM 20439 (92 %). The partial sequence of *bsh* for *B. bifidum* DSM 20456 exhibited the highest sequence similarity with *B. bifidum* S17 (99.6 %) and *B. bifidum* ATCC 15696 (99.4 %), and lower similarity (<99 %) with other analyzed strains from *B. bifidum* species. It is also noteworthy that 16S rDNA sequence similarities of all *B. bifidum* strains present in NCBI database were above 99 %. These results suggest that in some cases *bsh* may be a useful genetic marker for specific identification of analyzed bacteria even at the strain level. However, for more detailed evaluation of the utility of bile salt hydrolase-encoding gene for intraspecies differentiation of bifidobacteria further thorough studies involving a larger number of bacterial strains are necessary.

Highly conserved *bsh* sequences were characteristic for *B. breve* strains. Two analyzed members of this species had identical sequences for the partial *bsh* gene. Analysis of *bsh* sequences revealed a high degree of relatedness between *B. breve* strains and bifidobacteria from *B. longum* group, i.e., *B. longum* subsp. *longum* (99. 3 %), *B. longum* subsp. *suis* (98.5 %), and *B. longum* subsp. *infantis* (97.5 %). In contrast, *B. breve* was found to share a significantly lower similarity (below 87 %) with other analyzed species from the genus of *Bifidobacterium*. These results are in good agreement with the observations made by Ventura et al. [22] presenting *B. breve* as a closely related species with other bifidobacteria belonging to *B. longum* group.

The taxonomic position of *B. longum* subsp. *longum*, *B. longum* subsp. *infantis*, and *B. longum* subsp. *suis* was intensively explored in previous articles [14, 17]. The results obtained in this study also showed the very close phylogenetic relationship among these three taxa. However, clear differences were also evident. For *B. longum* subsp. *infantis* and *B. longum* subsp. *suis* used in our study, the partial sequences of *bsh* were found to share 97.7 and 98.7 % similarities, respectively, with *bsh* from type strain of *B. longum* subsp. *longum*. These results support the previous proposal to classify *B. longum* subsp. *longum*, *B. longum* subsp. *infantis*, and *B. longum* subsp. *suis* as three subspecies of *B. longum*.

The previous study revealed an extremely high 16S rDNA sequence similarity (99.5 %) between *B. catenulatum* and *B. pseudocatenulatum* [15]. Interestingly, our analysis of partial BSH gene sequences showed that *bsh* of *B.catenulatum* DSM 20224 was found to share only 93 % similarity with the sequence determined for *B. pseudocatenulatum* DSM 20439. The obtained results indicate the interspecies relationship between these two taxa and are consistent with the previous conclusion [24]. Our study also showed the utility of *bsh* for the genetic identification of *B. angulatum*, *B. gallicum*, and *B. pseudolongum*. The determined partial *bsh* sequences for these species revealed very low similarity with *bsh* from other bifidobacteria (below 73, 68, and 72 %, respectively). Moreover, the BSH DNA sequence similarity between the two subspecies of B. pseudolongum (*B. pseudolongum* subsp. *pseudolongum* and *B. pseudolongum* subsp. *globosum*) was only 90 %.

**Fig. 4:**
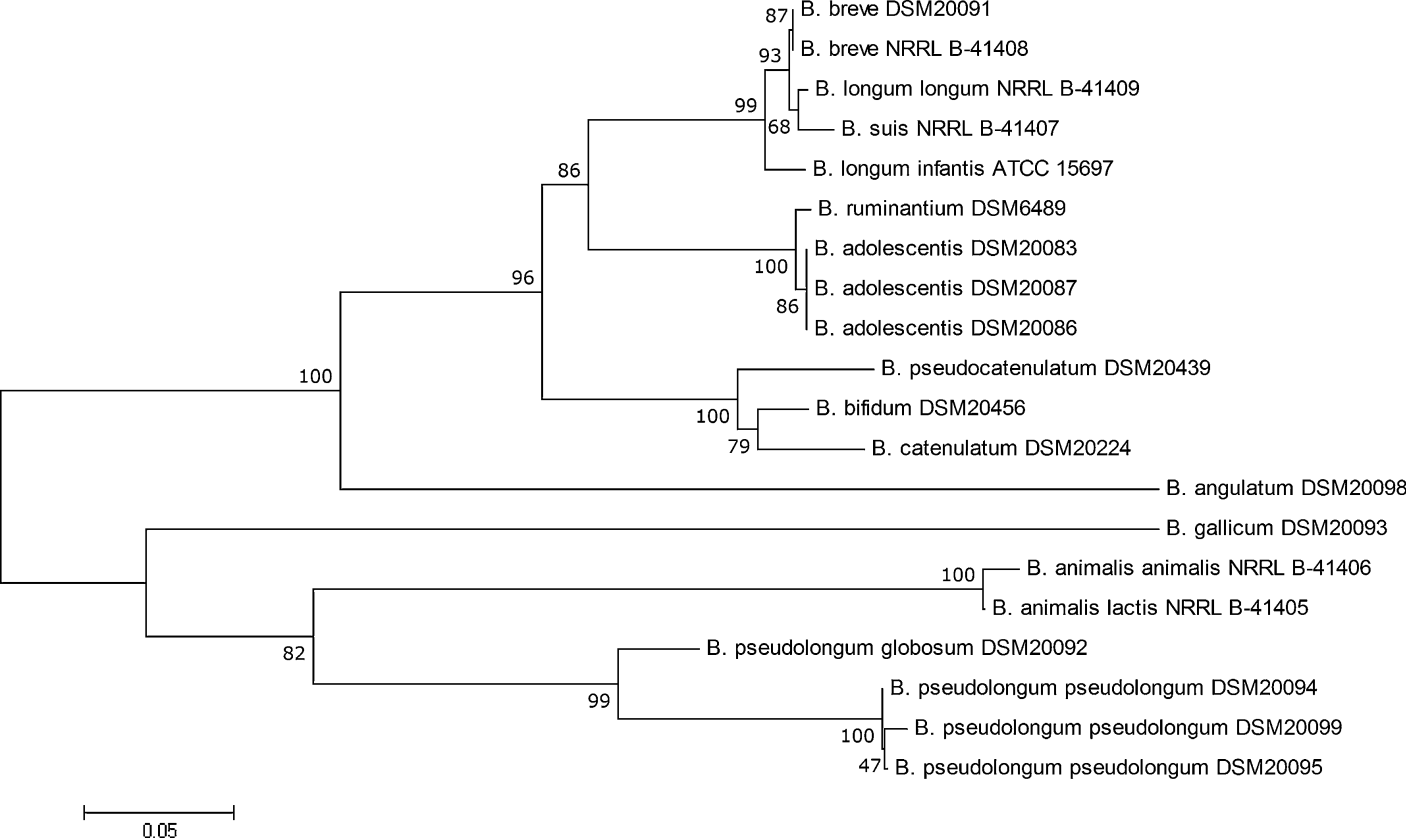
Phylogenetic trees based on partial sequences of *bsh* for bifidobacteria used in this study. Tree was constructed using the neighbor joining method from 1000 bootstrapping replicates

In general, the clustering tree for *bsh* sequences was similar to phylogenetic analysis based on 16S rRNA gene sequences obtained for bifidobacterial taxa used in this study (Fig. 4). However, clear differences were also evident. For the partial *bsh* sequence, the similarity among bifidobacterial species analyzed in this study ranged from 63.2 to 99.6 %. In comparison, 16S rRNA sequence similarity for *Bifidobacterium* species varied from 90.2 to 99.9 % [5]. Interestingly, very high similarities (above 99 %) were determined among bifidobacteria from *B. longum* group (*B. longum* and *B. breve*) and also for two species belonging to *B. adolescentis* group (*B. adolescentis* and *B. ruminantium*). These results are in good agreement with previous phylogenetic analysis of the genus *Bifidobacterium* using 16S rRNA sequences performed by Ventura et al. [22]. Further phylogenetic analysis should clarify the taxonomic position of these close-related taxa, i.e., the classification at subspecies level. The partial *bsh* gene sequences showed intraspecies variation up to 3 %; however, for two subspecies of *B. pseudolongum*, sequence difference reached more than 10 %. These results demonstrate that in some cases *bsh* sequences possess a definitely higher discriminatory power than the previously described genetic markers [5, 15, 22].

Based on the previously determined *bsh* sequences, internal primers were improved (Bif-bshE-3F 5′-TTCGGCCGYAAYCTYGAYTGG-3′ and Bif-bshF-3R 5′-TCGAYGACGATGSTDCG-3′) and then used for PCR screening and RT-PCR detection of transcription of *bsh* genes. As expected, the expression of *bsh* for all the analyzed bifidobacteria was confirmed by electrophoretic detection of an ~415-bp internal fragment of *bsh*.

## Conclusions

In summary, this study describes the use of *bsh* gene for the specific identification of human intestinal bifidobacteria. The phylogenetic analysis of the determined sequences revealed that the *bsh* genes showed significantly higher genetic variation than the bifidobacterial 16S rRNA sequences. Therefore, we conclude that bile salt hydrolase-encoding gene is a useful molecular marker for phylogenetic studies and specific identification of human fecal *Bifidobacterium* species.

## References

[1] Alschul SF, Gish W, Miller W, Myers EW, Lipman DJ (1990). Basic local alignment search tool. J Mol Biol.

[2] Chiang BL, Sheih YH, Wang LH, Liao CK, Gill HS (2000). Enhancing immunity by dietary consumption of probiotic lactic acid bacterium (*Bifidobacterium animalis* HN019): optimisation and definition of cellular immune responses. Eur J Clin Nutr.

[3] Fushinobu S (2010). Unique sugar metabolic pathways of bifidobacteria. Biosci Biotechnol Biochem.

[4] Haros M, Carlsson NG, Almgren A, Larsson-Alminger M, Sandberg AS, Andlid T (2009). Phytate degradation by human gut isolated *Bifidobacterium pseudocatenulatum* ATCC 27919 and its probiotic potential. Int J Food Microbiol.

[5] Jian W, Zhu L, Dong X (2001). New approach to phylogenetic analysis of the genus *Bifidobacterium* based on partial HSP60 gene sequences. Int J Syst Evol Microbiol.

[6] Jiang TA, Mustapha A, Savaiano DA (1996). Improvement of lactose digestion in humans by ingestion of unfermented milk containing *Bifidobacterium longum*. J Dairy Sci.

[7] Kim GB, Brochet M, Lee BH (2005). Cloning and characterization of a bile salt hydrolase (*bsh*) from *Bifidobacterium adolescentis*. Biotechnol Lett.

[8] Kim GB, Lee BH (2008). Genetic analysis of a bile salt hydrolase in *Bifidobacterium animalis* subsp. *lactis* KL61. J Appl Microbiol.

[9] Kim GB, Miyamoto CM, Meighen EA, Lee BH (2004). Cloning and characterization of the bile salt hydrolase genes (*bsh*) from *Bifidobacterium bifidum* strains. Appl Environ Microbiol.

[10] Krizova J, Spanova A, Rittich B (2008). RAPD and rep-PCR fingerprinting for characterization of *Bifidobacterium* species. Folia Microbiol.

[11] Leahy SC, Higgins DG, Fitzgerald GF, van Sinderen D (2005). Getting better with bifidobacteria. J Appl Microbiol.

[12] Masco L, Ventura M, Zink R, Huys G, Swings J (2004). Polyphasic taxonomic analysis of *Bifidobacterium animalis* and *Bifidobacterium lactis* reveals relatedness at the subspecies level: reclassification of *Bifidobacterium animalis* as *Bifidobacterium animalis* subsp. *animalis* subsp. nov. and *Bifidobacterium lactis* as *Bifidobacterium**animalis* subsp. *lactis* subsp. nov. Int J Syst Evol Microbiol.

[13] Matsuki T, Watanabe K, Tanaka R, Fukuda M, Oyaizu H (1999). Distribution of bifidobacterial species in human intestinal microflora examined with 16S rRNA-gene-targeted species-specific primers. Appl Environ Microbiol.

[14] Mattarelli P, Bonaparte C, Pot B, Biavati B (2008). Proposal to reclassify the three biotypes of *Bifidobacterium longum* as three subspecies: *Bifidobacterium longum* subsp. *longum* subsp. nov., *Bifidobacterium longum* subsp. *infantis* comb. nov. and *Bifidobacterium longum* subsp. *suis* comb. nov. Int J Syst Evol Microbiol.

[15] Miyake T, Watanabe K, Watanabe T, Oyaizu H (1998). Phylogenetic analysis of the genus *Bifdobacterium* and related genera based on 16S rDNA sequences. Microbiol Immunol.

[16] Saitou N, Nei M (1987). A neighbour-joining method: a new method for reconstructing phylogenetics trees. Mol Biol Evol.

[17] Sakata S, Kitahara M, Sakamoto M, Hayashi H, Fukuyama M, Benno Y (2002). Unification of *Bifidobacterium infantis* and *Bifidobacterium suis* as *Bifidobacterium longum*. Int J Syst Evol Microbiol.

[18] Tamura K, Dudley J, Nei M, Kumar S (2007). MEGA4: molecular evolutionary genetics analysis (MEGA) software version 4.0. Mol Biol Evol.

[19] Tanaka H, Hashiba H, Kok J, Mierau I (2000). Bile salt hydrolase of *Bifidobacterium longum*: biochemical and genetic characterization. Appl Environ Microbiol.

[20] Thompson JD, Higgins DG, Gibson TJ (1994). CLUSTAL W: improving the sensitivity of progressive multiple sequence alignment through sequence weighting, position specific gap penalties and weight matrix choice. Nucl Acids Res.

[21] Turroni F, van Sinderen D, Ventura M (2011). Genomics and ecological overview of the genus *Bifidobacterium*. Int J Food Microbiol.

[22] Ventura M, Canchaya C, Del Casale A, Dellaglio F, Neviani E, Fitzgerald GF, van Sinderen D (2006). Analysis of bifidobacterial evolution using a multilocus approach. Int J Syst Evol Microbiol.

[23] Ward P, Roy D (2005). Review of molecular methods for identification, characterization and detection of bifidobacteria. Lait.

[24] Zhu L, Li W, Dong X (2003). Species identification of genus *Bifidobacterium* based on partial HSP60 gene sequences and proposal of *Bifidobacterium thermacidophilum* subsp. *porcinum* subsp. nov. Int J Syst Evol Microbiol.

